# Implementation of electronic patient-reported outcome measures in hemodialysis: Findings from a matched cohort study

**DOI:** 10.1177/11297298261446694

**Published:** 2026-05-13

**Authors:** João Pedro Barros, João Almeida Fonseca, Rui Pinto, Jorge Pratas, Ana Luísa Correia, Alcides Pereira, Beatriz Queirós, Rui André Sousa, Charmaine E Lok, Ricardo-Cruz Correia

**Affiliations:** 1 Programme in Health Data Science, University of Porto, Porto, Portugal; 2 NephroCare, Fresenius Medical Care Portugal, Portugal; 3 CINTESIS@RiSE, MEDCIDS, University of Porto, Porto, Portugal; 4 Hemodialysis Unit Nephrology Unidade local de Saúde de Coimbra, Coimbra, Portugal; 5 Nephrology Department, ULS do Tâmega e Sousa, CHTS, Penafiel, Portugal; 6 Toronto General Hospital, Toronto, ON, Canada; 7 University of Toronto, Toronto, ON, Canada; 8 CINTESIS, MEDCIDS, University of Porto, Porto, Portugal

**Keywords:** Patient reported outcome measures, kidney failure, chronic, renal dialysis, kidney failure with replacement therapy, implementation, quality of life, treatment outcome

## Abstract

**Background:**

Integrating electronic patient-reported outcome measures (ePROMs) into routine hemodialysis (HD) care offers a way to enhance patient-centered decision-making.

**Methods:**

This prospective matched cohort study involved adults on in-center HD followed for 6 months. The intervention group completed monthly ePROMs (SF-VAQ and SF-36v2), while the control group received standard care. Outcomes included changes from baseline in Vascular Access (VA) related interventions, hospitalizations, SF-VAQ domain scores, and Health-Related Quality of Life (HRQoL).

**Results:**

Integrating ePROMs did not significantly impact the frequency of VA-related interventions or hospitalizations during the study period. Significant improvements were observed in the SF-VAQ domains, including reductions in perceived VA-related burden in the Physical (*p* = 0.002), Social (*p* = 0.001), and Dialysis-Related Complication (*p* = 0.001) domains. The Physical Component Summary (PCS) and Mental Component Summary (MCS) of the SF-36v2 also improved significantly (PCS: *p* = 0.001; MCS: *p* = 0.000). A total of 31 (55%) of patients in the ePROM group achieved the Minimal Clinically Important Difference.

**Conclusions:**

Implementation of an ePROM-guided care pathway is feasible and associated with improved patient-reported well-being and reduced VA-related burden. While these results suggest a benefit, the absence of longitudinal process data in the control arm means further research is required to distinguish ePROM-induced changes from standard care evolution.

## Background

Hemodialysis (HD) treatments require reliable vascular access (VA),^1^ which can be an arteriovenous fistula (AVF), prosthetic arteriovenous graft (AVG), or a central venous catheter (CVC). In addition to being indispensable, VA has been regarded as the Achilles heel for patients receiving HD due to their associated complications, such as infections, hematoma, aneurysm and pseudoaneurysm, stenosis and thrombosis, requiring often inconvenient and painful interventions.^2^

As such, there is a need to implement a patient-centered care approach to VA that balances the patient’s preferences, access function, survival, and potential complications, especially for elderly patients.^3^ The emphasis on patient-centered care has led to the implementation of a fresh approach in healthcare, aiming to achieve the “right access, for the right patient, at the right time, for the right reasons.”^4^ Promoting patient’s choices and preferences in the VA field has become increasingly adopted,^5^ tailored to individual patients based on their clinical needs, values, and preferences.^6^

Nonetheless, Patient-reported Outcomes (PRO)—that extend beyond biochemical markers and include outcomes that matter most to patients, including pain and Health-Related Quality of Life (HRQoL)—remain under-assessed in HD clinical practice.^7,8^ Specific Patient-reported Outcome Measure (PROMs) have been developed for VA to support a patient-centered care pathway and improve quality of care. The most frequently reported outcomes are VA function, infection, and maturation.^9^ Patients have shown an increasing interest in the use of PRO, even with infrequent assessment periods, particularly on the impact of interventions on VA comfort or satisfaction.^7^

This study aims to describe the implementation process and evaluate the impact of electronic patient-reported outcome measures (ePROMs), with a particular focus on the Short-Form Vascular Access Questionnaire (SF-VAQ), which captures patient-reported perceptions of pain, bruising, bleeding, functional limitations, and overall satisfaction related to VA. The impact of integrating ePROMs into VA care will be measured by the changes in VA-related interventions and hospitalizations, SF-VAQ domains, and HRQoL using the 36-Item Short Form Health Survey version 2 (SF-36v2). The study will also determine the Minimal Clinically Important Difference (MCID) for the SF-VAQ and compare patient-reported experiences across different VA types and cannulation techniques.

## Methods

This prospective matched cohort study was conducted across two private dialysis centers. Participants receiving ePROMs matched in a 1:2 ratio with unexposed controls based on key demographic and clinical variables. The study comprised two analytical components. First, a between-group comparison evaluated the effect of ePROMs exposure on clinical outcomes recorded for all patients (hospitalization and VA interventions), and a within-group longitudinal analysis examined the evolution of SF-VAQ and SF-36v2 scores, predictors of VA satisfaction, and MCID achievement exclusively within the ePROM intervention arm, as these instruments were not administered to the standard of care group. This second component, therefore, constitutes a single-arm, repeated-measures design (Supplementary File 1).

Eligible participants were adults aged 18 years or older who had been receiving HD for at least 3 months, exhibited no or only minimal cognitive impairment, and provided written informed consent. Cognitive ability was assessed using the 6-item Cognitive Impairment Test (6CIT-P).^10^ Group differences at baseline were assessed using *t*-tests for continuous variables and chi-square χ^2^ tests for categorical variables.

Participants were followed for 6 months, during which data were electronically collected. The exposed group received conventional HD treatment supplemented with ePROM, while the unexposed group received standard HD treatment without exposure to ePROMs or any additional interventions.

Electronic data capture reflects the preferred method reported by patients and aligns with recommendations to integrate ePROMs within Electronic Health Record (EHR) systems.^11^ While the process was designed for independent completion, minimal assistance was provided by nurses as needed; in this cohort, 24.6% of participants (*n* = 15) required nursing support to complete the assessments. To ensure the clinical utility of the collected data, a structured feedback loop was established wherein ePROM results were systematically reviewed by the dialysis nursing and clinical teams. These findings served as a focal point during multidisciplinary meetings, where they were triangulated with clinical observations to inform care planning. When relevant, ePROM data were shared directly with patients during treatment sessions to facilitate shared decision-making and patient engagement. This process led to the implementation of specific practice changes, most notably the refinement of cannulation techniques and broader VA management protocols. By embedding these activities into existing clinical workflows, the study successfully transitioned ePROMs from a data collection tool into a routine component of dialysis care, ultimately driving patient-centered improvements in VA outcomes.

### PROMS

The PROMs collected in the exposed group were the Portuguese versions of the SF-VAQ^12^ and the SF-36v2.^13^ The SF-VAQ is a 13-item PRO measure that assesses satisfaction with the VA in patients on HD. It includes four domains: VA Overall Satisfaction (VA-OS; 1 item), Physical Symptoms (VA-PS; 4 items), Social Functioning (VA-SF; 4 items), and Dialysis-Related Complications (VA-DRC; 4 items). Each item is rated on a 7-point Likert scale (1 = highly satisfied, 7 = highly dissatisfied). Summing the 12 evaluative items from the VA-PS, VA-SF, and VA-DRC domains yields the SF-VAQ total score, ranging from 12 to 84, with lower totals indicating greater satisfaction.^14^

The SF-36v2 is a validated 36-item questionnaire that measures HRQoL across eight domains: Physical Functioning (PF), Role Physical (RP), Bodily Pain (BP), General Health (GH), Vitality (VT), Social Functioning (SF), Role Emotional (RE), and Mental Health (MH). It produces two summary scores: Physical Component Summary (PCS) and Mental Component Summary (MCS). Each domain is scored from 0 to 100, with higher scores indicating better health status.^15^ The Portuguese version of the scale and the calculation of scores for the PCS and MCS were used.^16^ Formal authorization and licensing for the use of this instrument were obtained from QualityMetric (License Number: QM056071).

### Calculation of clinically meaningful change

This study, using ePROM over the follow-up period, determined the MCID of the implemented SF-VAQ. The MCID represents the smallest change in the PROMs that the patient perceives as beneficial for treatment decisions, emphasizing a patient-centered outcomes approach, where observed changes are not only statistically significant but also meaningful to patients.^17,18^

MCID was calculated using distribution-based methods,^19^ defined as 0.5 times the Standard Deviation (SD) of baseline scores (T0).^20^

### Statistical analysis

The statistical modeling strategy was defined according to the operational nature of each outcome and the study’s structural design, concurrently addressing the longitudinal repeated measures and the matched-pairs framework. Continuous variables and domain scores from the SF-VAQ and SF-36v2 were summarized using means and standard deviation (SD), with baseline comparisons between the ePROM intervention group and the matched control group performed via paired Student’s *t*-tests for normally distributed data, or the Mann–Whitney *U* and Kruskal–Wallis tests for independent groups, depending on the results of Shapiro-Wilk and Levene’s tests. To estimate the effect of ePROM implementation on primary clinical outcomes, such as hospitalization and VA interventions, Odds Ratios (OR) were calculated using Generalized Linear Mixed-effects Models (GLMMs) adjusted for age, gender, and HD vintage; these models utilized a random intercept to account for the dependency within the matched-pair design.

The longitudinal evolution of ePROM scores, including VA satisfaction and MCID achievement, was analyzed using Linear Mixed-effects Models (LMMs) and GLMMs, respectively. To account for intra-patient correlation, repeated measures were nested within participants including random intercept, with LMMs fitted using the Restricted Maximum Likelihood (REML) algorithm to provide unbiased variance estimates. Furthermore, the specific impact of SF-VAQ dimensions on cannulation problems and VA interventions was assessed using the GLMMs function with random effects, while multicollinearity among predictors was monitored using the Variance Inflation Factor (VIF), with values exceeding five indicating concern. Patient satisfaction with VA and achievement of the MCID were modeled using Mixed-Effects Random Forest (MERF) models and complementary LMMs and GLMMs. In both analyses, MERF models incorporated random effects at the patient level to account for repeated measures, with hyperparameters tuned for optimal performance including 1000 trees, 15 variables per split, 10 optimization iterations, and Ornstein–Uhlenbeck stochastic modeling of random effects. SHapley Additive exPlanations (SHAP) plots were used to visualize the relative contribution of each ePROM variable, providing both global and individual-level feature importance for patient satisfaction and MCID outcomes. The top five SHAP-ranked predictors from each MERF model were subsequently carried forward as fixed effects in the inferential LMMs and GLMMs, reducing dimensionality and limiting the risk of fitting noise.

A two-stage framework was employed. First, MERF was used exclusively for exploratory feature selection, as it accounts for within-patient clustering and captures non-linear relationships without pre-specification.^21,22^ Variable importance was quantified using SHAP values.^23^ Second, the top-ranked features were carried forward into LMM and GLMM models for confirmatory inference. All reported coefficients, confidence intervals, and *p*-values derive from these conventional models.

To evaluate model generalization and guard against overfitting, a strict temporal out-of-sample validation was adopted: for each patient with seven complete observations, the first five time points were allocated to the training set and the remaining two to the test set, ensuring that all predictions were assessed on future, unseen data. Out-of-bag (OOB) samples provide an unbiased internal estimate of generalization error at each iteration. Performance was compared between training and test sets using *R*^2^, Root Mean Square Error (RMSE), and Mean Absolute Error (MAE) for continuous outcomes, and accuracy (AUC), sensitivity, and specificity for binary outcomes. Missing data were handled based on variable type: stable demographics used the most recent recorded value, while longitudinal PROMs were handled via Multiple Imputation by Chained Equations (MICE) using Predictive Mean Matching (PMM) to prevent variance underestimation. Data were analyzed using RStudio software (version 4.4.2) with the mice, longituRF, lme4, and lmerTest packages.

## Results

### Comparative analysis of exposed and unexposed groups

Of the 61 patients in the ePROM group who met the study’s inclusion criteria, 47 (77.05%) were male, with a mean age of 69.65 (±11.08) years and an HD vintage of 42.19 (±27.64) months (Table 1). Of the 61 patients enrolled at baseline (T0), 59 (96.7%) completed T1 and T2, 59 (96.7%) T3, 58 (95.1%) T4, 57 (93.4%) T5 and T6. Overall attrition from T0 to the final assessment (T6) was 6.6% (4 patients), and 56 patients (91.8%) completed all ePROM assessments.

**Table 1.** Exposed and unexposed groups allocation variables (ratio 1:2).

Allocation variables	Unexposed group	ePROM group	*p*-Value
Sample (*n*)	122	61	—
Gender male (*n*, %)	80 (65.57%)	47 (77.05%)	0.178
Age (mean ± SD)	71.94 (±12.79)	69.65 (±11.08)	0.214
Vascular access (*n*, %)			0.157
Arteriovenous fistula (AVF)	104 (85.24%)	49 (80.32%)	
Arteriovenous graft (AVG)	5 (4.10%)	7 (11.48%)	
Central venous catheter (CVC)	13 (10.66%)	5 (8.20%)	
ESKD etiology (ICD 10) (*n*, %)			0.124
E10.2	17 (13.93%)	4 (6.56%)	
N18.5	11 (9.02%)	6 (9.84%)	
N18.9	43 (35.25%)	14 (22.95%)	
Others	51 (41.80%)	37 (60.65%)	
Bartel score (mean, ±SD)	100.00 (±0.00)	99.96 (±0.25)	0.321
Charlson Comorbidity Index Score (mean, ±SD)	4.23 (±2.08)	3.34 (±1.32)	**0.000**
Hemodialysis vintage (mean months, ±SD)	68.16 (±60.23)	42.19 (±27.64)	**0.000**

SD: standard deviation; CVC: central venous catheter; AVF: arteriovenous fistula; AVG: arteriovenous graft; ESRD: end-stage renal disease; ICD-10: International Classification of Diseases, 10th Revision.

Values in bold indicate statistically significant differences (p < 0.05).

The patient allocation in the exposed and unexposed group was similar, except that patients who received ePROM had fewer comorbidities and had shorter HD vintage (Table 1). During the study, two patients in the ePROM group dropped out (Supplementary File 2). No statistically significant differences were found between the two groups related to VA interventions (angiography, angioplasty, angioplasty with stent, thrombectomy, clinical consult, or surgical procedure). To account for baseline clinical discrepancies between the ePROM and control groups, a multivariate logistic regression analysis was performed for hard clinical outcomes (Table 2). The distribution of events related to VA interventions, Mortality and Hospitalization (*p* = 1.0 vs *p* = 0.83 vs *p* = 0.50) are reported in Supplementary File 2.

**Table 2.** Vascular access interventions and hospitalization associated with ePROM implementation.

Study variables	Univariate OR	95% CI	*p*-Value	Multivariate OR	95% CI	*p*-Value
Vascular access interventions	0.95	0.30–2.98	0.929	0.87	0.28–2.68	0.811
Hospitalization	0.59	0.22–1.57	0.290	0.52	0.20–1.33	0.170

Odds ratios (OR) and 95% confidence intervals (CI) for factors associated with ePROM implementation based on univariate and multivariate logistic regression analyses. The multivariate model includes adjustments for Age, Gender, and Hemodialysis vintage.

### Outcomes in the exposed (ePROM) group

Compared with baseline (T0), 6-month (T6), SF-VAQ results demonstrated significant differences based on access type and cannulation technique (Supplementary File 3). AVG patients reported the highest burden for pain (3.140 ± 1.983), bleeding (2.302 ± 2.199), and hospitalization worry (4.884 ± 1.762), while AVF was associated with the highest swelling (2.501 ± 1.955). Conversely, CVC users reported superior aesthetic scores (4.231 ± 1.861) but faced the greatest challenges regarding bathing (4.731 ± 2.376) and general care (3.500 ± 2.177). Among cannulation techniques, Multiple Single Cannulation Technique (MuST) yielded the highest satisfaction (6.070 ± 0.828; *p* = 0.002), whereas Rope-Ladder (RL) and Buttonhole (BT) reported the greatest care difficulty and bruising, respectively (*p* < 0.05). The improvement in patient outcomes followed a structured clinical pathway mediated by the ePROM intervention. At baseline, the ePROM feedback identified significant burdens related to VA, reflected in high scores for Dialysis Complications (VA-DRC: 12.8 ± 4.91) and BP (BP: 66.1 ± 30.8).

In response to these patient-reported alerts and ongoing clinical evaluation, targeted adjustments in cannulation techniques were implemented. This led to a strategic shift away from the more traumatic Area (AR) technique (decreasing from 25 to 21 patients) toward vessel-preserving and standardized approaches, such as the BT and MuST techniques, which increased from 11 to 12 and 5 to 7 patients, respectively (Table 3).

**Table 3.** Longitudinal evolution of Health-Related Quality-of-Life scores (SF-36v2) and Vascular-Access scores (SF-VAQ) from baseline (T0) to 6 months follow-up (T6).

Dimensions	T0			T1			T2			T3			T4			T5			T6			*p*-Value
	Mean (SD)	VA	AVT	Mean (SD)	VA	VAT	Mean (SD)	VA	VAT	Mean (SD)	VA	VAT	Mean (SD)	VA	VAT	Mean (SD)	VA	VAT	Mean (SD)	VA	VAT	
*SF-VAQ*																						
Overall satisfaction with Vascular Access (VA-OS)	5.54 ± 1.31	AVF—49, AVG—7, CVC—5	AR—25, RL—16, BT—11, MuST—5	5.29 ± 1.49	AVF—48, AVG—6, CVC—5	AR—24, RL—15, BT—11, MuST—5	5.34 ± 1.35	AVF—49, AVG—6, CVC—4	AR—23, RL—14, BT—12, MuST—6	5.61 ± 1.33	AVF—50, AVG—6, CVC—3	AR—23, RL—14, BT—13, MuST—6	5.78 ± 1.27	AVF—49, AVG—6, CVC—3	AR—22, RL—14, BT—12, MuST—7	5.88 ± 1.06	AVF—48, AVG—6, CVC—3	AR—22, RL—13, BT—12, MuST—7	5.41 ± 1.37	AVF—48, AVG—6, CVC—3	AR—21, RL—14, BT—12, MuST—7	0.788
Physical Function (VA-PF)	9.84 ± 5.67			9.98 ± 5.20			10.5 ± 4.91			8.09 ± 4.07			7.48 ± 4.46			7.53 ± 4.34			7.50 ± 4.36			**0.002**
Social Function (VA-SF)	9.87 ± 4.65			9.00 ± 4.63			10.5 ± 4.52			8.71 ± 4.81			7.45 ± 4.46			7.54 ± 4.25			7.63 ± 3.98			**0.001**
Dialysis Complications (VA-DRC)	12.8 ± 4.91			10.9 ± 3.46			12.0 ± 3.32			11.3 ± 4.28			8.55 ± 4.11			9.07 ± 4.18			9.67 ± 4.10			**0.001**
SF-VAQ Total Score	32.5 ± 11.2			29.9 ± 9.58			33.0 ± 9.53			28.1 ± 11.2			23.5 ± 10.8			24.2 ± 10.3			24.7 ± 9.42			**0.000**
Cannulation Problems (*n*)	10			7			8			3			12			11			12			
SF-36v2																						
Physical Functioning (PF)	39.4 ± 22.8	AVF—49, AVG—7, CVC—5	AR—25, RL—16, BT—11, MuST—5	48.6 ± 23.1	AVF—48, AVG—6, CVC—5	AR—24, RL—15, BT—11, MuST—5	39.3 ± 23.3	AVF—49, AVG—6, CVC—4	AR—23, RL—14, BT—12, MuST—6	48.5 ± 27.1	AVF—50, AVG—6, CVC—3	AR—23, RL—14, BT—13, MuST—6	53.2 ± 24.4	AVF—49, AVG—6, CVC—3	AR—22, RL—14, BT—12, MuST—7	50.7 ± 22.4	AVF—48, AVG—6, CVC—3	AR—22, RL—13, BT—12, MuST—7	53.6 ± 21.4	AVF—48, AVG—6, CVC—3	AR—21, RL—14, BT—12, MuST—7	**0.000**
Role-Physical (RP)	44.5 ± 24.6			44.4 ± 21.8			43.9 ± 24.5			53.9 ± 28.8			53.7 ± 26.7			49.6 ± 28.7			51.8 ± 27.9			**0.005**
Bodily Pain (BP)	66.1 ± 30.8			56.5 ± 28.7			62.5 ± 33.5			70.8 ± 27.4			70.3 ± 26.0			74.9 ± 30.1			73.0 ± 30.5			**0.042**
General Health (GH)	38.9 ± 14.0			37.4 ± 14.3			36.3 ± 15.9			36.1 ± 15.6			41.6 ± 13.8			38.4 ± 17.1			37.3 ± 16.0			0.936
Vitality (VT)	49.0 ± 19.0			47.2 ± 213.4			44.3 ± 15.8			46.5 ± 20.8			47.3 ± 20.6			49.7 ± 17.1			55.7 ± 19.0			**0.010**
Social Functioning (SF)	74.8 ± 17.5			67.6 ± 19.8			74.6 ± 21.1			74.4 ± 14.0			73.3 ± 15.1			80.6 ± 19.0			85.0 ± 17.6			**0.000**
Role-Emotional (RE)	57.8 ± 25.3			61.3 ± 26.5			65.4 ± 27.7			75 ± 20.9			73.4 ± 19.6			84.8 ± 21.6			88.5 ± 21.2			**0.000**
Mental Health (MH)	69.3 ± 19.0			66.4 ± 13.9			68.3 ± 15.5			71.1 ± 19.7			70.8 ± 19.3			75.4 ± 20.7			76.8 ± 21.1			**0.026**
Physical Component Summary (PCS)	46.9 ± 8.34			50.6 ± 8.69			45.8 ± 5.05			50.8 ± 8.48			55.2 ± 7.80			50.1 ± 8.63			50.9 ± 8.35			**0.001**
Mental Component Summary (MCS)	49.1 ± 9.28			44.4 ± 7.54			50.0 ± 7.29			49.1 ± 7.47			46.4 ± 7.41			54.0 ± 7.81			57.4 ± 9.45			**0.000**

VA: Vascular Access; VAT: Vascular Access cannulation technique; CVC: Central Venous Catheter; AVF: Arteriovenous Fistula; AVG: Arteriovenous Graft; RL: Rope-Ladder; BT: Buttonhole; AR: Area; MuST: Multiple Single Cannulation Technique.

Intermediate assessment points are labeled T2, T3, T4, T5, and T6. Each line (or column) represents one SF-VAQ and SF-36v2 dimension: Overall Satisfaction (VA-OS), Physical Symptoms (VA-PS), Social Functioning (VA-SF), and Dialysis-Related Complications (VA-DRC), Physical Functioning (PF), Role-Physical (RP), Bodily Pain (BP), General Health (GH), Vitality (VT), Social Functioning (SF), Role-Emotional (RE), Mental Health (MH), Physical Component Summary (PCS), and Mental Component Summary (MCS), and, the four SF-VAQ domains: VA Overall Satisfaction (VA-OS), Physical Symptoms (VA-PF), Social Functioning (VA-SF), and Dialysis-Related Complications (VA-DRC).

Scores are expressed as mean and standard deviation (SD). Statistically significant differences (*p* < 0.05) are shown in bold.

This transition in clinical practice directly corresponds with the significant longitudinal improvements observed at 6 months. Specifically, there was a marked reduction in access-related complications (VA-DRC improved to 9.67 ± 4.10; *p* = 0.001) and physical function limitations (VA-PF: *p* = 0.002). These physical improvements were accompanied by a robust enhancement in psychological and social well-being, as evidenced by significant increases in MH (MH: *p* = 0.026), RE (RE: *p* = 0.000), and the MCS (MCS: 49.1–57.4; *p* = 0.000). Collectively, these data demonstrate that the ePROM system functioned as a dynamic clinical tool, where patient feedback triggered technical refinements that ultimately enhanced both VA stability and overall HRQoL (Table 3).

The reported nursing cannulation problems also influence the VA-PF (β = 0.812, 95% CI: 0.080, 1.544, *p* = 0.030), VA-DRC (β = 0.844, 95% CI: 0.127, 1.562, *p* = 0.022), SF-VAQ Total score (β = 1.948, 95% CI: 0.243, 3.653, *p* = 0.026; Supplementary File 4).

**Figure 1.**
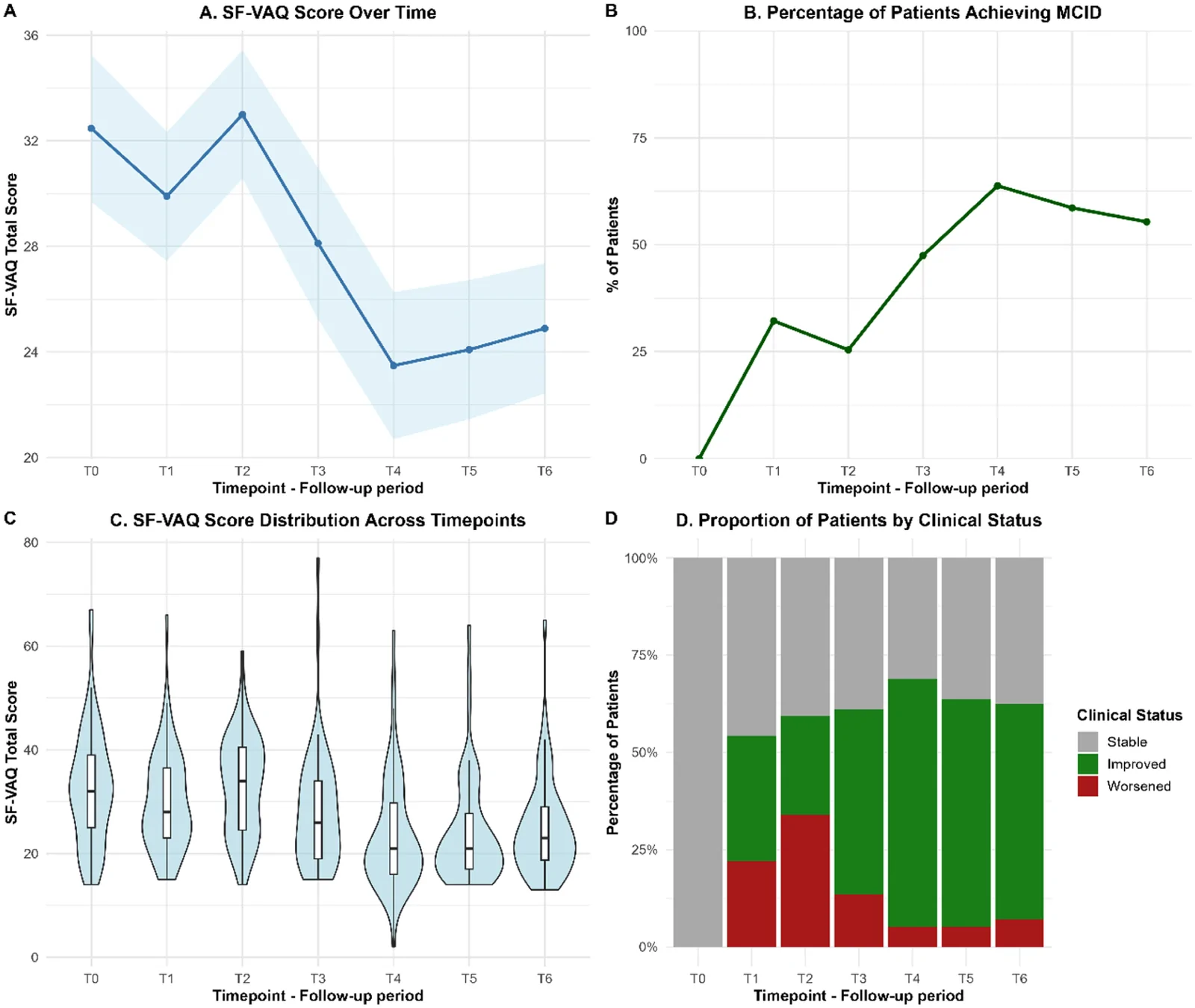
Minimal Clinically Important Difference (MCID) evolution over study timepoints, and SF-VAQ Total Score evolution.

Importantly, the proportion of patients achieving the MCID progressively increased over time. The baseline MCID was 5.58, and by 6 months, 31 (55%) of patients had achieved this threshold of improvement (Table 4). Additionally, an overall improvement in the SF-VAQ total score was observed across study time points, with a growing number of patients reaching the established MCID (Figure 1).

**Table 4.** Minimal Clinically Important Difference (MCID) calculation and proportion of patients achieving the MCID in SF-VAQ scores across study follow-up timepoints.

Measure	Timepoint	Mean	SD	MCID
SF-VAQ total score	T0	32.475	11.15	5.58
Patients achieve MCID evolution over a 6-month follow-up period				
Timepoint	Improved (*n*/%)	No change (*n*/%)	Worsened (*n*/%)	
T0	0 (0%)	61 (100%)	0 (0%)	
T1	19 (32%)	27 (46%)	13 (22%)	
T2	15 (25%)	24 (41%)	20 (34%)	
T3	28 (47%)	23 (39%)	8 (14%)	
T4	37 (64%)	18 (31%)	3 (5%)	
T5	34 (59%)	21 (36%)	3 (5%)	
T6	31 (55%)	21 (38%)	4 (7%)	

SD: standard deviation.

Percentage of patients achieving the Minimal Clinically Important Difference (MCID) at each follow-up time point during the study period. Values are presented as percentages of the total number of patients assessed at each time point.

SHAP summary plots illustrate the relative contribution and directional impact of key SF-VAQ features on predictions of overall VA satisfaction and MCID achievement (Supplementary File 5). The MERF models, used exclusively for SHAP-based feature selection, exhibited the training-to-test performance divergence typical of flexible ensemble methods. For VA Satisfaction, the training *R*^2^ of 0.659 decreased to 0.270 on the test set; however, the OOB *R*^2^ (0.348) closely approximated test performance, confirming that internal bootstrap validation provides a reliable generalization estimate. For MCID, the model achieved a training AUC of 0.985 versus a test AUC of 0.796 (training accuracy = 0.932; test accuracy = 0.714), with OOB estimation explaining 63.64% of variance. The features identified by the MERF models were carried forward into inferential models, which demonstrated robust generalization on held-out data.

Multivariable analysis (Model 1) demonstrated stable generalization, with nearly identical prediction errors between training (RMSE = 1.012) and test sets (RMSE = 1.032), and a test-to-train *R*^2^ ratio of 0.74. Pain (β = −0.346, 95% CI −0.496 to −0.196; *p* = 0.000) and difficulty with bathing (β = −0.251, 95% CI −0.413 to −0.089; *p* = 0.000) were identified as significant negative predictors of overall VA satisfaction. VA vintage, age, and concerns related to hospitalization were not significantly associated with satisfaction (Table 5).

**Table 5.** Linear and generalized linear mixed model estimates for vascular access satisfaction and MCID using top predictors identified by the MERF model.

Model	Variable	Coefficient (β)	95% CI (β)	*p*-Value
Model 1—Vascular Access Overall Satisfaction	Q4 Pain	−0.346	−0.496, −0.196	0.000
	VA Vintage	−0.027	−0.235, 0.181	0.802
	Age	−0.088	−0.296, 0.120	0.410
	Q11 Bathing	−0.251	−0.413, −0.089	0.000
	Q14 Hospitalized	0.021	−0.127, 0.169	0.783
	Variable	OR	95% CI (OR)	*p*-Value
Model 2—Minimal Clinically Important Difference	Q9 VA Appearance	0.097	0.030, 0.315	0.000
	Q14 Hospitalized	0.158	0.055, 0.451	0.000
	Q4 Pain	0.270	0.106, 0.684	0.005
	Q15 VA Longevity	0.178	0.065, 0.486	0.000
	Q7 Bruising	0.300	0.124, 0.726	0.007

Summarize the results from the linear mixed-effects model (LMM) for vascular access (VA) satisfaction and the generalized linear mixed-effects model (GLMM) for predicting achievement of the Minimal Clinically Important Difference (MCID). Both models include random intercepts for patient to account for repeated measurements. For the LMM, Beta coefficients, 95% confidence intervals, and *p*-values are presented for the top five predictors identified through SHAP importance ranking. For the GLMM, Beta estimates, odds ratios (OR), and corresponding 95% confidence intervals are shown. Negative Beta values and OR < 1 indicate decreased satisfaction or reduced likelihood of achieving MCID, respectively. Q# indicates the corresponding item number from the Short Form Vascular Access Questionnaire (SF-VAQ). Model performance (train/test) was summarized for the LMM using *R*^2^, RMSE, and MAE (*R*^2^ = 0.4062/0.3008; RMSE = 1.0128/1.0325; MAE = 0.7784/0.8218) and for the GLMM using accuracy and AUC at a 0.5 threshold (Accuracy = 0.9679/0.8393; AUC = 0.9906/0.9307).

Model 2 maintained strong discriminative ability on held-out data (test AUC = 0.930; train AUC = 0.990), with balanced sensitivity (84.4%) and specificity (83.3%) confirming its reliability for clinical prediction. Poorer VA appearance, greater concern regarding hospitalization, higher pain levels, reduced perceived access longevity, and increased bruising were all independently associated with a lower likelihood of achieving MCID (all *p* < 0.05; Table 5).

## Discussion

Our 6-month follow-up found that implementing the ePROM system and our patient-reported data feedback, were associated with significant improvements in patient satisfaction, VA-related symptom burden and an increase in HRQoL. However, there was no reduction in VA associated interventions or all-cause hospitalizations. This finding aligns with reports from recent patient-centered trials where short intervention duration, the rapid progression of underlying risk factors, and already high standards of VA care limited any additional impact of PRO use on hard clinical events. Our findings echo a recent Dutch ePROM study in dialysis patients, which concluded that focusing on improving patients’ experience and symptom management may be more fruitful than expecting immediate reductions in hospitalizations rates.^24^

The adoption of self-report measurement tools, such as PROMs, can improve communication, detect deterioration in patient condition, and assess the impact of healthcare delivery, promoting healthcare-shared decisions between patients and stakeholders.^25^ For example, early in the study, patient feedback via the SF-VAQ highlighted issues like swelling and pain at cannulation sites. In response, our team introduced targeted VA management adjustments (e.g. more intensive fistula monitoring and alternative needling techniques), which coincided with improvements in patient-reported VA comfort and a reduction in local complications. This iterative intervention illustrates how PROMs data can prompt clinical changes aimed at preventing access deterioration, even if the overall rate of formal surgical interventions did not differ in the short term.

These findings prompted targeted adjustments to our VA management protocol: systematic troubleshooting of fistula sites and adoption of alternative cannulation techniques designed to minimize trauma.

Patient satisfaction with their VA improved over the course of the study, particularly among those using AVFs. At baseline, participants reported low satisfaction alongside considerable pain, bruising and swelling at cannulation sites. These issues were most pronounced in patients with AVGs or CVCs. By the end of follow-up, mean VA satisfaction scores had increased, coinciding with a decline in reported pain intensity and local access complications. Our results mirror prior findings that satisfaction levels differ markedly by VA type: patients with AVFs tend to be more satisfied with their access than those with grafts or catheters.^12,14,26^ In our cohort, AVF users indeed reported the highest satisfaction, whereas AVG users experienced more frequent pain, bleeding, bruising and anxiety about access longevity, and CVC users voiced more difficulties with daily activities (e.g. bathing) due to their access. These patterns are consistent with the literature, where AVFs are generally preferred by patients for comfort and durability.^14^ AVGs, while sometimes necessary, are known to incur more needling pain and complications,^27^ explaining their lower satisfaction scores. Likewise, CVCs, though avoiding needles, can impede lifestyle (showering, swimming) and carry infection risks, diminishing patient contentment.^28^ Beyond access type, our analysis highlighted which specific problems drive dissatisfaction. The SF-VAQ domains indicated that physical symptoms (especially cannulation-related pain and bleeding) and dialysis-related complications at the access were major contributors to poor satisfaction. In fact, using a MERF, we found that “pain during needling” were the top predictors of the overall VA satisfaction score. This aligns with qualitative reports that patients often tolerate their fistula or graft well until it causes pain or visible harm.^28^ The most commonly cited physical complaints related to VA in other studies include swelling, bruising, and bleeding, precisely the issues that our intervention sought to mitigate. By actively troubleshooting patient-reported problems—such as adjusting cannulation technique or rotating needling sites to minimize trauma—we observed a tangible improvement in satisfaction scores. Notably, prior research has demonstrated that higher VA-related symptom burden and complications correlate with worse psychosocial outcomes. Patients reporting greater VA pain, frequent infiltrations, or VA infections also report significantly lower QoL and higher depression levels.^29,30^

During the 6-month intervention, patients in exposed ePROM group demonstrated notable improvements. The serial assessments showed a decrease in VA-related symptom burden alongside an increase in HRQoL. Specifically, scores in all domains of the SF-VAQ improved significantly from baseline at the end of 6 months: physical symptoms, social functioning, dialysis-related complications, and the total SF-VAQ score all improved (i.e. lower score). Concurrently, patients’ HRQoL scores on the SF-36v2 improved across multiple domains. We observed statistically significant gains in PF, RP, BP, VT, SF, RE, and MH, as well as in both summary measures (PCS and MCS). These findings suggest that integration of ePROM-derived data into routine clinical decision-making may have facilitated more responsive symptom management and psychosocial support, contributing to a measurable improvement in patients’ perceived health status. However, in the absence of parallel longitudinal outcome monitoring in the control arm, these improvements should be interpreted as hypothesis-generating, and the independent contribution of ePROM feedback cannot be definitively established.

Also, we observed a significant inverse association between overall VA-related distress and mental well-being: as satisfaction with VA improved (lower SF-VAQ total score) the SF-36v2 MCS score increased. This relationship underscores how closely patients’ mental health is tied to the burden of their VA, with an alleviating VA problems, such as needling pain or fear of VA failure, likely contributed to reduced stress and improved emotional health for our participants.

This relationship between VA satisfaction and patients’ HRQoL has been seen in other studies where higher VA dissatisfaction levels, for example, due to infection, pain, difficulty with use, complications, physical symptoms were linked to depression, and negatively associated QoL levels.^30,31^ The use of an ePROM approach allowed us to detect and respond to issues that standard labs and exams might overlook—translating into better PRO.^32^

Our study supports that by overcoming barriers to PRO measurement and implementation, attention to HRQoL domains affected by VA, give patients a voice in achieving their desired VA outcomes with a patient-centered emphasis on VA functionally.^32,33^ The Standardized Outcomes in Nephrology–Hemodialysis (SONG-HD) initiative has proposed a core outcome set for VA, which reflects the perspectives and priorities for HD patients.^5^ These include outcomes that are reliable, valid, and responsive, such as the capacity to receive uninterrupted HD with an adequate dose without the need for unplanned interventions. Additional core domains comprise the ability to cannulate the VA without complications, pain, hospitalization events, VA replacement, infection, steal syndrome, or aneurysm formation. Crucially, these outcomes capture the patient’s experience of care, including satisfaction with VA and the broader impact on QoL.^5^

Lastly, the baseline MCID improved over the follow-up period, underscoring the significance of PRO and its impact on clinical decision making to implement evidence-based interventions.^17,34^ The attainment of the MCID throughout follow-up highlights that it’s not just clinical change, but a patient-perceived improvement substantial enough to influence real-world function and care satisfaction.

HD intervention studies frequently demonstrate statistically significant group-level improvements, yet individual-level MCID responder analyses often reveal modest proportions of patients achieving meaningful change.^35^ By targeting VA—the patient’s “lifeline” and a primary source of anxiety and physical discomfort—interventions address what matters most to patients’ daily experience. This focused approach may drive a more pronounced shift in perceived well-being, translating group-level gains into higher individual MCID attainment rates.

We identified several features impacting the odds of MCID achievement, as pain, appearance, and bruising, and specifically related to cannulation technique. MuST was associated with higher levels of satisfaction with less bruising and pain compared with other cannulation techniques (RL, BT, or AR). These findings are compatible with other studies that reported on VA physical complications, which include swelling, bruising, and bleeding.^27^ Indeed, the MuST technique has previously shown to have fewer bruising complications compared to the RL or BT technique.^36^ A recent meta-analysis demonstrated that MuST can significantly reduce bruising and infiltration complications compared to both RL and BT methods.^37^

The divergence between the VA Satisfaction (Model 1) and MCID Achievement (Model 2) highlights that patient-reported satisfaction often captures different nuances than objective clinical success. Specifically, it frames the MCID as a critical threshold for “clinically meaningful improvement,” distinguishing incremental, statistically detectable changes from the more substantial shifts that patients truly perceive as important.^38^

Overall, the differences between techniques underscore the value of individualizing cannulation to reduce complications important to patients. Our results support the growing advocacy for patient-tailored VA care with the cannulation technique definition and adjusted to patient-specific anatomy and tolerance, may offer better outcomes than a one-size-fits-all approach. By aligning the cannulation strategy with patient preference and access condition, it is possible to improve both satisfaction and clinical metrics like VA longevity.

The study has some limitations. The two study groups had differences related to HD vintage and the Charlson Comorbidity Score. These variables can influence VA dimensions, particularly complications associated with the early stages of HD.^39^ Also assessing HD VA requires long-term, longitudinal data to accurately measure “hard” outcomes like primary failure and secondary patency, rather than relying on short-term markers.^7^ This exploratory study was not powered to detect significant differences in hard clinical outcomes. Results regarding hospitalization and VA interventions should be interpreted as hypothesis-generating signals rather than definitive evidence of efficacy.

The use of MERF for feature selection in a sample of 61 patients (410 observations) warrants caution. Although MERF appropriately handles hierarchical data structures and avoids the instability of conventional stepwise methods in correlated datasets,^22,23,40^ it was used exclusively as an exploratory screening tool—all inferential conclusions derive from conventional mixed-effects models. The feature rankings identified through SHAP-based analysis^24^ should be regarded as hypothesis-generating and require external validation in larger, independent cohorts.

This study also highlights the importance of integrating PROMs in routine clinical care to improve patients’ experiences and reduce any negative experiences. However, persistent challenges to implement PROMs tools include clinical engagement, patient support, feasible technology, and getting feedback, with nurses playing a vital role in collecting Patient-Generated Health Data.^25^ This task is additional to their patient care responsibilities at a time when nurses and technicians are at risk of burn out. Another limitation to ascertainment is, the monthly recall period which may affect data accuracy, as there are no established guidelines for an optimal recall timeframe for patients. Lastly, patients’ responses may be influenced if they did not experience immediate symptom relief or noticeable improvement in their condition.^41^ The MCID estimates based on distribution-derived methods are limited in that they do not incorporate patient perspectives and may not accurately represent clinically meaningful change.^42^

Nevertheless the implementation of ePROM in this study supports health measurement tools as essential for screening patients’ needs and experiences, as well as for improving HRQoL.^43,44^

Future studies should explore the cost-effectiveness of ePROM interventions in HD, as the sustainability of these initiatives will depend on demonstrating both clinical and economic value. In summary, this study demonstrates potential benefits of implementing ePROMs in routine VA care, while also underscoring key operational challenges. These include the need to integrate ePROM collection into existing dialysis workflows, ensure staff engagement and training in interpreting and acting on the data, support patients with varying levels of digital literacy, and establish efficient systems for data review and feedback. Addressing these through thoughtful study design, improved stakeholder engagement, and timely use of PROMs data will be essential. By continuing to refine this patient-centered approach, we move closer to achieving high-quality, individualized dialysis care that listens to, and acts upon, what matters most to patients.

### Conclusion

The integration of ePROMs into routine vascular access care has the potential to improve patient satisfaction and HRQOL, while also revealing key implementation challenges that must be addressed for long-term sustainability. These findings support the role of PROMs as complementary, patient-centered tools that augment traditional clinical markers, enhance patient engagement, and facilitate shared decision-making.

## Supplemental Material

sj-docx-1-jva-10.1177_11297298261446694 – Supplemental material for Implementation of electronic patient-reported outcome measures in hemodialysis: Findings from a matched cohort study Supplemental material, sj-docx-1-jva-10.1177_11297298261446694 for Implementation of electronic patient-reported outcome measures in hemodialysis: Findings from a matched cohort study by João Pedro Barros, João Almeida Fonseca, Rui Pinto, Jorge Pratas, Ana Luísa Correia, Alcides Pereira, Beatriz Queirós, Rui André Sousa, Charmaine E Lok and Ricardo-Cruz Correia in The Journal of Vascular Access

sj-docx-2-jva-10.1177_11297298261446694 – Supplemental material for Implementation of electronic patient-reported outcome measures in hemodialysis: Findings from a matched cohort study Supplemental material, sj-docx-2-jva-10.1177_11297298261446694 for Implementation of electronic patient-reported outcome measures in hemodialysis: Findings from a matched cohort study by João Pedro Barros, João Almeida Fonseca, Rui Pinto, Jorge Pratas, Ana Luísa Correia, Alcides Pereira, Beatriz Queirós, Rui André Sousa, Charmaine E Lok and Ricardo-Cruz Correia in The Journal of Vascular Access

sj-docx-3-jva-10.1177_11297298261446694 – Supplemental material for Implementation of electronic patient-reported outcome measures in hemodialysis: Findings from a matched cohort study Supplemental material, sj-docx-3-jva-10.1177_11297298261446694 for Implementation of electronic patient-reported outcome measures in hemodialysis: Findings from a matched cohort study by João Pedro Barros, João Almeida Fonseca, Rui Pinto, Jorge Pratas, Ana Luísa Correia, Alcides Pereira, Beatriz Queirós, Rui André Sousa, Charmaine E Lok and Ricardo-Cruz Correia in The Journal of Vascular Access

sj-docx-4-jva-10.1177_11297298261446694 – Supplemental material for Implementation of electronic patient-reported outcome measures in hemodialysis: Findings from a matched cohort study Supplemental material, sj-docx-4-jva-10.1177_11297298261446694 for Implementation of electronic patient-reported outcome measures in hemodialysis: Findings from a matched cohort study by João Pedro Barros, João Almeida Fonseca, Rui Pinto, Jorge Pratas, Ana Luísa Correia, Alcides Pereira, Beatriz Queirós, Rui André Sousa, Charmaine E Lok and Ricardo-Cruz Correia in The Journal of Vascular Access

sj-docx-5-jva-10.1177_11297298261446694 – Supplemental material for Implementation of electronic patient-reported outcome measures in hemodialysis: Findings from a matched cohort study Supplemental material, sj-docx-5-jva-10.1177_11297298261446694 for Implementation of electronic patient-reported outcome measures in hemodialysis: Findings from a matched cohort study by João Pedro Barros, João Almeida Fonseca, Rui Pinto, Jorge Pratas, Ana Luísa Correia, Alcides Pereira, Beatriz Queirós, Rui André Sousa, Charmaine E Lok and Ricardo-Cruz Correia in The Journal of Vascular Access
