# Supplementary material for: Implementation of electronic patient-reported outcome measures in hemodialysis: Findings from a matched cohort study
Source: J Vasc Access. 2026 May 13;27(5):2272–83. doi: 10.1177/11297298261446694 (PMC13547692; doi:10.1177/11297298261446694)
Supplement: Supplementary file 1 [file sj-docx-1-jva-10.1177_11297298261446694.docx]

**Supplementary File 1 –** Flowchart of the study protocol illustrating the recruitment process, 1:2 allocation, 6-month follow-up period, and the specific metrics used for between-group and within-group clinical analysis.
