# Supplementary material for: Implementation of electronic patient-reported outcome measures in hemodialysis: Findings from a matched cohort study
Source: J Vasc Access. 2026 May 13;27(5):2272–83. doi: 10.1177/11297298261446694 (PMC13547692; doi:10.1177/11297298261446694)
Supplement: Supplementary file 2 [file sj-docx-2-jva-10.1177_11297298261446694.docx]

**Supplementary File 2 –** Distribution of variables across Unexposed and ePROM group

| **Variables** | **Unexposed group**  **(n = 122)** | **ePROM group**  **(n=61)** | **p-value*** |
| --- | --- | --- | --- |
| Vascular Access Interventions (n) | 33 | 16 | 1.00 |
| Intervention Type (n)  Angiography  Angioplasty  Angioplasty with stent  Thrombectomy  Consult  Surgical Procedure  Vascular Access Faillure | 12  14  4  4  2  5  1 | 6  5  1  1  7  3  1 | ------------------ |
| Total Interventions (n) | 42 | 24 |  |
| Mortality (n) | 8 | 2 | 0.50 |
| Hospitalization (n) | 19 | 8 | 0.83 |

***Legend****: Vascular access interventions, vascular access failure, mortality, and hospitalization events among ePROM and unexposed hemodialysis patients group, during the six-month follow-up period.
Data are presented as the total number of events observed in each group. The ePROM group received conventional HD treatment supplemented with ePROM implementation, while the non-exposed group received standard HD treatment without ePROM. “Vascular Access Interventions” includes all procedures performed to maintain or restore vascular access function (angiography, angioplasty, angioplasty with stent, thrombectomy, clinical consult, or surgical procedure). “Vascular Access Failure” refers to permanent loss of access function requiring creation of a new access site.*
