# Supplementary material for: Implementation of electronic patient-reported outcome measures in hemodialysis: Findings from a matched cohort study
Source: J Vasc Access. 2026 May 13;27(5):2272–83. doi: 10.1177/11297298261446694 (PMC13547692; doi:10.1177/11297298261446694)
Supplement: Supplementary file 3 [file sj-docx-3-jva-10.1177_11297298261446694.docx]

**Supplementary File 3 –** Short-Form Vascular Access (SF-VAQ) score distribution across different Vascular Access and Cannulation Techniques

| **Domain** | **Question** | **n obs** | **Overall** | | **n obs** | **AVF** | | **n obs** | **AVG** | | **n obs** | **CVC** | | **p-value** | **Canulation Technique** | | | | | | | | **p-value** |
| --- | --- | --- | --- | --- | --- | --- | --- | --- | --- | --- | --- | --- | --- | --- | --- | --- | --- | --- | --- | --- | --- | --- | --- |
|  |  |  | **Mean** | **SD±** |  | **Mean** | **SD±** |  | **Mean** | **SD±** |  | **Mean** | **SD±** |  | **AR** | | **RL** | | **MST** | | **BT** | |  |
| **Vascular Access Overall satisfaction (VA-OS)** |  |  |  |  |  |  |  |  |  |  |  |  |  |  | **Mean** | **SD±** | **Mean** | **SD±** | **Mean** | **SD±** | **Mean** | **SD±** |  |
|  | (Q3) - I am satisfied with my vascular access | 410 | 5.59 | 1.29 | 341 | 5.610 | 1.298 | 43 | 5.535 | 0.935 | 26 | 5.423 | 1.629 | 0.592 | 5.612 | 1.256 | 5.079 | 1.589 | 6.070 | 0.828 | 5.771 | 1.213 | **0.002** |
| **Physical Function (VA-PF)** | (Q4) - During the past 4 weeks I was bothered by pain associated with my vascular access | 410 | 2.66 | 1.92 | 340 | 2.707 | 1.941 | 43 | 3.140 | 1.983 | 26 | 1.308 | 0.618 | **0.000** | 2.750 | 2.027 | 2.746 | 1.769 | 2.372 | 1.915 | 2.771 | 1.934 | 0.571 |
|  | (Q5) - During the past 4 weeks I was bothered by bleeding with my vascular access | 410 | 1.65 | 1.43 | 340 | 1.622 | 1.335 | 43 | 2.302 | 2.199 | 26 | 1.038 | 0.196 | **0.023** | 1.658 | 1.505 | 1.635 | 1.126 | 1.814 | 1.577 | 1.446 | 0.966 | 0.433 |
|  | (Q6) - During the past 4 weeks I was bothered by swelling associated with my vascular access | 410 | 2.40 | 1.92 | 340 | 2.501 | 1.955 | 43 | 2.419 | 1.955 | 26 | 1.154 | 0.543 | **0.000** | 2.737 | 2.068 | 2.397 | 1.880 | 2.000 | 1.952 | 2.410 | 1.760 | 0.110 |
|  | (Q7) - During the past 4 weeks I was bothered by bruising associated with my vascular access | 410 | 2.01 | 1.65 | 340 | 2.023 | 1.671 | 43 | 2.395 | 1.801 | 26 | 1.231 | 0.652 | **0.016** | 1.980 | 1.609 | 2.429 | 1.820 | 1.535 | 1.222 | 2.048 | 1.814 | **0.036** |
| **Social function (VA-SF)** | (Q8) - During the past 4 weeks my access interfered with my daily activities (e.g. work, social, leisure activities or other regular daily activities) | 410 | 2.53 | 1.82 | 340 | 2.478 | 1.782 | 43 | 2.651 | 2.034 | 26 | 3.038 | 1.990 | 0.257 | 2.546 | 1.932 | 2.746 | 1.694 | 2.186 | 1.435 | 2.301 | 1.709 | 0.380 |
|  | (Q9) - During the past 4 weeks I was bothered by the appearance of my vascular access | 410 | 3.04 | 2.08 | 340 | 2.977 | 2.089 | 43 | 2.860 | 2.042 | 26 | 4.231 | 1.861 | **0.008** | 3.257 | 2.218 | 3.032 | 1.967 | 3.047 | 2.214 | 2.386 | 1.759 | 0.032 |
|  | (Q10) - During the past 4 weeks my access interfered with my sleep | 410 | 1.57 | 1.23 | 341 | 1.540 | 1.204 | 43 | 1.977 | 1.640 | 26 | 1.346 | 0.745 | 0.270 | 1.605 | 1.282 | 1.571 | 1.058 | 1.465 | 1.260 | 1.434 | 1.139 | 0.450 |
|  | (Q11) - During the past 4 weeks my access caused me problems when bathing or showing | 410 | 1.57 | 1.44 | 341 | 1.299 | 0.910 | 43 | 1.814 | 0.816 | 26 | 4.731 | 2.376 | **0.000** | 1.197 | 0.609 | 1.508 | 1.230 | 1.070 | 0.258 | 1.446 | 1.212 | 0.090 |
| **Dialysis Complications (VA-DRC)** | (Q12) - During the past 4 weeks my vascular access had problems (i.e. didn’t work properly) | 410 | 1.76 | 1.52 | 341 | 1.663 | 1.351 | 43 | 2.209 | 2.088 | 26 | 2.346 | 2.208 | 0.216 | 1.572 | 1.221 | 1.683 | 1.148 | 1.651 | 1.602 | 1.819 | 1.571 | 0.301 |
|  | (Q13) - During the past 4 weeks my vascular access was difficult to care for (i.e. dressings, trying to keep access clean and protected) | 410 | 2.11 | 1.59 | 341 | 1.924 | 1.385 | 43 | 2.767 | 2.114 | 26 | 3.500 | 2.177 | **0.000** | 1.783 | 1.276 | 2.397 | 1.487 | 1.628 | 1.092 | 1.976 | 1.554 | **0.008** |
|  | (Q14) - During the past 4 weeks I was worried about being hospitalized because of problems with my access | 410 | 3.00 | 2.07 | 341 | 2.783 | 1.990 | 43 | 4.884 | 1.762 | 26 | 2.769 | 2.160 | **0.000** | 2.651 | 2.040 | 3.175 | 1.828 | 2.628 | 1.865 | 2.807 | 2.069 | 0.118 |
|  | (Q15) - During the past 4 weeks I was worried about how long my vascular access will last | 410 | 3.77 | 1.36 | 341 | 3.792 | 1.326 | 43 | 3.953 | 1.379 | 26 | 3.231 | 1.704 | 0.085 | 3.914 | 1.446 | 3.810 | 1.120 | 3.605 | 1.237 | 3.651 | 1.282 | 0.377 |

**Legend:** Distribution of SF-VAQ scores by Vascular Access type and cannulation technique. Rows list every questionnaire item (Q3 – Q14) together with its domain label: Vascular Access Overall Satisfaction (VA-OS); Vascular Access Physical Symptoms (VA-PF); Vascular Access Social Function (VA-SF); Vascular Access Dialysis-Related Complications (VA-DRC). Columns show mean and Standard Deviation (SD) for each Vascular Access category, Central Venous Catheter (CVC), Arteriovenous Fistula (AVF) and Arteriovenous Graft (AVG), and for each cannulation technique Rope-Ladder (RL), Buttonhole (BT), Area (AR) and Multiple Single Cannulation Technique  (MuST). Items are scored on a 7-point Likert scale.
