# Supplementary material for: Implementation of electronic patient-reported outcome measures in hemodialysis: Findings from a matched cohort study
Source: J Vasc Access. 2026 May 13;27(5):2272–83. doi: 10.1177/11297298261446694 (PMC13547692; doi:10.1177/11297298261446694)
Supplement: Supplementary file 4 [file sj-docx-4-jva-10.1177_11297298261446694.docx]

**Supplementary File 4 –** Short-Form Vascular Access (SF-VAQ) scores association with Vascular Access Intervention and Canulation Problems

| **Vascular Access Intervention** | | | |
| --- | --- | --- | --- |
| **SF-VAQ Dimensions** | **Coefficient (β)** | **95% CI (β)** | **p-value** |
| Vascular Access Overall Satisfaction (VA-OS) | 0.408 | -0.248 – 1.064 | 0.223 |
| Physical Function (VA-PF) | 0.876 | -1.408 – 3.161 | 0.453 |
| Social Function (VA-PF) | 0.310 | - 1.786 – 2.405 | 0.772 |
| Dialysis Related Complications (VA-DRC) | 2.166 | -0.049 – 4.380 | 0.056 |
| SF-VAQ Total Score | 3.110 | -2.204 – 8.425 | 0.252 |
| **Canulation Problems** | | | |
| **SF-VAQ Dimensions** | **Coefficient (β)** | **95% CI (β)** | **p-value** |
| Overall satisfaction with Vascular Access (VA-OS) | 0.059 | -0.130 – 0.272 | 0.584 |
| Physical Function (VA-PF) | 0.812 | 0.080 – 1.544 | **0.030** |
| Social Function (VA-SF) | 0.301 | -0.373 – 0.975 | **0.382** |
| Dialysis Related Complications (VA-DRC) | 0.844 | 0.127 – 1.562 | **0.022** |
| SF-VAQ Total Score | 1.948 | 0.243 – 3.653 | **0.026** |

**Legend:** Association of Vascular Intervention and Canulation Problems with the SF-VAQ different domains. Vascular Access Overall Satisfaction (VA-OS); Vascular Access Physical Symptoms (VA-PF); Vascular Access Social Function (VA-SF); Vascular Access Dialysis-Related Complications (VA-DRC). The table shows regression coefficients (β), 95% confidence intervals (CI), and p-values for each predictor. Statistically significant differences (p < 0.05) are shown in bold.
