# Supplementary material for: Implementation of electronic patient-reported outcome measures in hemodialysis: Findings from a matched cohort study
Source: J Vasc Access. 2026 May 13;27(5):2272–83. doi: 10.1177/11297298261446694 (PMC13547692; doi:10.1177/11297298261446694)
Supplement: Supplementary file 5 [file sj-docx-5-jva-10.1177_11297298261446694.docx]

**Supplementary File 5** - SHAP summary plots for the MERF models predicting Vascular Access overall satisfaction (left) and Minimal Clinically Important Difference (MCID) achievement (right). Each point represents an individual observation, with color indicating the feature value (blue = low, red = high). Features are ordered by their mean absolute SHAP contribution, reflecting their global importance in the model.

**Legend** - SHAP-based feature importance from the Mixed-Effects Random Forest (MERF) models. The VA Satisfaction MERF achieved a training R² of 0.659, RMSE = 0.768, MAE = 0.607, and a test R² of 0.270, RMSE = 1.055 and MAE = 0.794, with an OOB R² of 0.348 approximating test performance. The MCID MERF yielded a training AUC of 0.985 and a test AUC of 0.796 (training accuracy = 0.932; test accuracy = 0.714; OOB variance explained = 63.64%).
